# CDK9 inhibitors selectively target estrogen receptor-positive breast cancer cells through combined inhibition of *MYB* and *MCL-1* expression

**DOI:** 10.18632/oncotarget.6997

**Published:** 2016-01-24

**Authors:** Partha Mitra, Ren-Ming Yang, James Sutton, Robert G. Ramsay, Thomas J. Gonda

**Affiliations:** ^1^ School of Pharmacy, University of Queensland, Brisbane, QLD, Australia; ^2^ Novartis Institute for Biomedical Research, Emeryville, CA, USA; ^3^ Peter MacCallum Cancer Centre, Melbourne, VIC, Australia; ^4^ Sir Peter MacCallum Department of Oncology, University of Melbourne, Melbourne, VIC, Australia

**Keywords:** CDK9 inhibitors, MYB, breast cancer, transcription pausing, apoptosis

## Abstract

Our previous studies showed that *MYB* is required for proliferation of, and confers protection against apoptosis on, estrogen receptor-positive (ER^+ve^) breast cancer cells, which are almost invariably also MYB^+ve^. We have also shown that *MYB* expression in ER^+ve^ breast cancer cells is regulated at the level of transcriptional elongation and as such, is suppressed by CDK9*i*. Here we examined the effects of CDK9*i* on breast cancer cells and the involvement of *MYB* in these effects. ER^+ve^ breast cancer cell lines including MCF-7 were much more sensitive (> 10 times) to killing by CDK9*i* than ER^−ve^/MYB^−ve^ cells. Moreover, surviving cells showed a block at the G2/M phase of the cell cycle. Importantly, ectopic *MYB* expression conferred resistance to apoptosis induction, cell killing and G2/M accumulation. Expression of relevant MYB target genes including *BCL2* and *CCNB1* was suppressed by CDK9 inhibition, and this too was reversed by ectopic *MYB* expression. Nevertheless, inhibition of BCL2 alone either by *MYB* knockdown or by ABT-199 treatment was insufficient for significant induction of apoptosis. Further studies implied that suppression of *MCL-1*, a well-documented target of CDK9 inhibition, was additionally required for apoptosis induction, while maximal levels of apoptosis induced by CDK9*i* are likely to also involve inhibition of *BCL2L1* expression. Taken together these data suggest that MYB regulation of *BCL2* underlies the heightened sensitivity of ER^+ve^ compared to ER^−ve^ breast cancer cells to CDK9 inhibition, and that these compounds represent a potential therapeutic for ER^+ve^ breast cancers and possibly other *MYB*-dependent cancers.

## INTRODUCTION

*MYB* encodes a transcription factor that plays key roles in normal function and cancers of the hematopoietic system, mammary and colonic epithelium and certain other tissues [1], [2]. It has been known for some time that *MYB* is highly expressed in estrogen receptor-positive (ER^+ve^) breast cancer [3], which reflects the fact that *MYB* is a direct target of estrogen/ER signaling (ER). More recently our laboratories have shown that *MYB* is required for the proliferation of breast cancer cells [4], contributes to suppression of apoptosis and differentiation, and is involved in the modulation of epithelial-mesenchymal transition [5, 6]. Importantly we also demonstrated that *MYB* is required for mammary tumour formation and/or progression in mouse models, and is frequently upregulated in metastases [7, 8].

The anti-apoptotic role of *MYB* in breast cancer was not immediately apparent since shRNA-mediated knockdown did not induce significant apoptosis by itself. However, MYB knockdown greatly enhanced the sensitivity of breast cancer cells to several chemical agents, an effect mediated (at least in part) by the MYB target gene *BCL2*, since knockdown of the latter mimicked the sensitisation effects of *MYB* knockdown [5].

Given these findings we have proposed that *MYB* may be a valuable and broadly-applicable therapeutic target in breast cancer [9]. As a transcription factor, though, MYB itself is not currently considered to be readily “druggable”. However, our work on the regulation of *MYB* expression in breast cancer has suggested an alternate approach to suppress *MYB* activity. Specifically it has become apparent that *MYB* expression is frequently regulated by a transcriptional elongation block imposed by a motif in the first intron comprised of a stem-loop-forming sequence followed by a poly(dT) tract (SL-dT) [10]. We have further shown that in ER^+ve^ breast cancer cells, this block is overcome by estrogen-stimulated ER binding in the vicinity of the SL-dT region [11] and direct ER-mediated recruitment of the elongation-promoting P-TEFb complex [12]. P-TEFb functions by phosphorylation, through its kinase component CDK9, of substrates including specific serine residues (Ser2) in the C-terminal domain of RNA polymerase II. A number of CDK9 inhibitors (CDK9*i*) are available and in breast cancer cells, such inhibitors can re-impose the block to *MYB* transcriptional elongation and suppress *MYB* expression [12].

While there have been several studies on the effects of CDK9*i* on breast cancer cells [13-15], relatively few relevant targets, other than *MCL-1,* have been widely reported. Here we have examined, in the present report, the potential of CDK9*i* to suppress the proliferation and/or viability of ER^+ve^ breast cancer cells through the inhibition of *MYB* expression. We show that CDK9i can induce apoptosis and inhibit proliferation of ER^+ve^/MYB^+ve^ breast cancer cells, while MYB^−ve^ breast cancer cells are much less sensitive to these compounds. Furthermore ectopic *MYB* expression can protect ER^+ve^ breast cancer cells against CDK9*i*, suggesting that the effects of the latter are at least partly due to *MYB* down-regulation. However, mechanism of apoptosis induction by CDK9*i* is more complex, appearing to involve direct inhibition of *MCL1* expression as well as suppression, through decreased *MYB* expression, of BCL2 levels.

## RESULTS

### CDK9*i* selectively downregulate *MYB* expression by imposing transcriptional pausing

**Figure 1:**
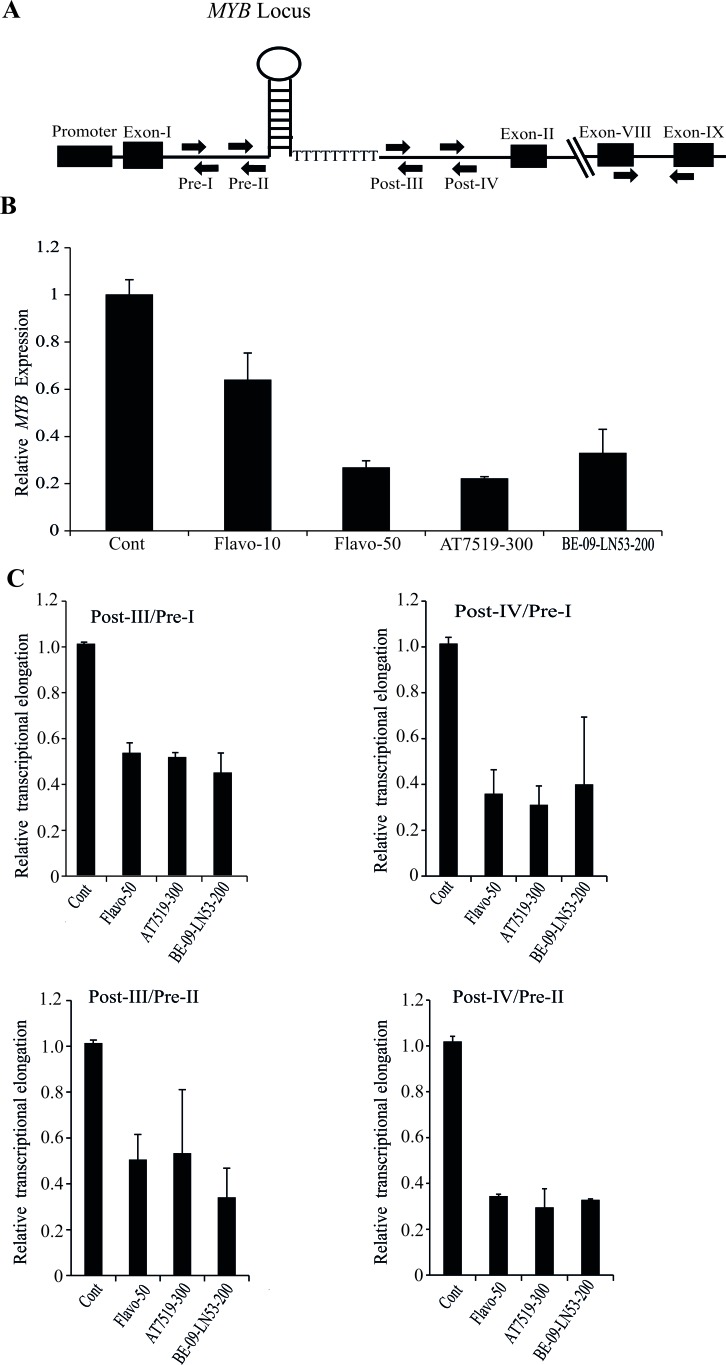
Transcription of MYB is attenuated at the pausing site within intron-I in breast cancer cells by CDK9i **A.** Schematic diagram of human c-MYB gene showing the promoter, intron-1 containing a stem-loop forming region followed by poly dT tract (SL-dT motif). Locations of primers used for the detection of intronic transcripts (Pre-I, Pre-II and Post-III, Post-IV), and for the mature transcript (exons 8 and 9) are indicated. **B.** CDK9i selectively downregulate the expression of MYB. MCF-7 cells were incubated with different CDK9i as shown (Flavo-, Flavopiridol; AT7519; BE-09-LN53;) for 4h. The concentrations of each drug in nM are shown next to the name of the drug. Expression of MYB was normalized using β-actin gene expression as an internal control. **C.** CDK9i induced pausing at the SL-dT region. MCF-7 cells were treated with the indicated CDK9i for 4h and qPCRs were performed using the indicated intronic primers. Relative expression of intronic transcripts measured by Pre- and Post- primer pairs was determined using a β-actin intronic transcript as an internal control. The degree of pausing is expressed as a ratio of relative expression of Pre to Post-transcription, normalised to untreated controls. All qPCRs (*n* ≥ 3) were performed in triplicate with error bars indicating standard deviation (SD).

We tested a number of recently developed CDK*i* and compared these with Flavopiridol for their ability to suppress *MYB* expression and impose an elongation block at the *MYB* SL-dT region. These compounds included AT7519, which is a multi-CDK inhibitor with a very low IC50 (<10nM) for CDK9, and is currently in phase-II clinical trials for several cancers [17-20]. We also used a new inhibitor, BE-09-LN53, which has a substantially greater specificity for CDK9 compared to other CDKs [21]. MCF-7 cells were treated with these compounds, along with Flavopiridol, for 4h, following which we determined the expression of mature *MYB* mRNA. It is clear from Figure 1B that expression of *MYB* is downregulated by all these drugs. Full dose-response studies of each drug (See Supplementary Figure S1A-E), and confirmation of inhibition of RNA Pol II Ser2 phosphorylation by AT7519 are shown in Supplementary Figure S1.

Next we examined the correlation of drug-mediated downregulation of *MYB* with respect to transcriptional pausing. For this purpose we used our previously-described [12] primer pairs Pre-I, Pre-II, Post-III and Post-IV (Figure 1A) to detect transcription before or after the SL-dT region. As seen in Figure 1C, the ratios of post- to pre- transcripts were at least two fold lower with each CDK9*i* compared to untreated controls. Thus, Flavopiridol, AT7519 and BE-09-LN53 all inhibit *MYB* expression by suppressing transcriptional elongation. This transcriptional inhibition by CDK9*i* also reflected in dose dependent downregulation of MYB expression when extracts of MCF-7 cells, treated with AT7519 or Flavopiridol at different dosages, western blotted with anti MYB antibody (Supplementary Figure 2A and 2B).

### ER^+ve^*MYB*^+ve^ cell lines are more sensitive than ER^−Ve^ *MYB*^−Ve^ cells to CDK9 inhibitors-induced apoptosis

**Figure 2:**
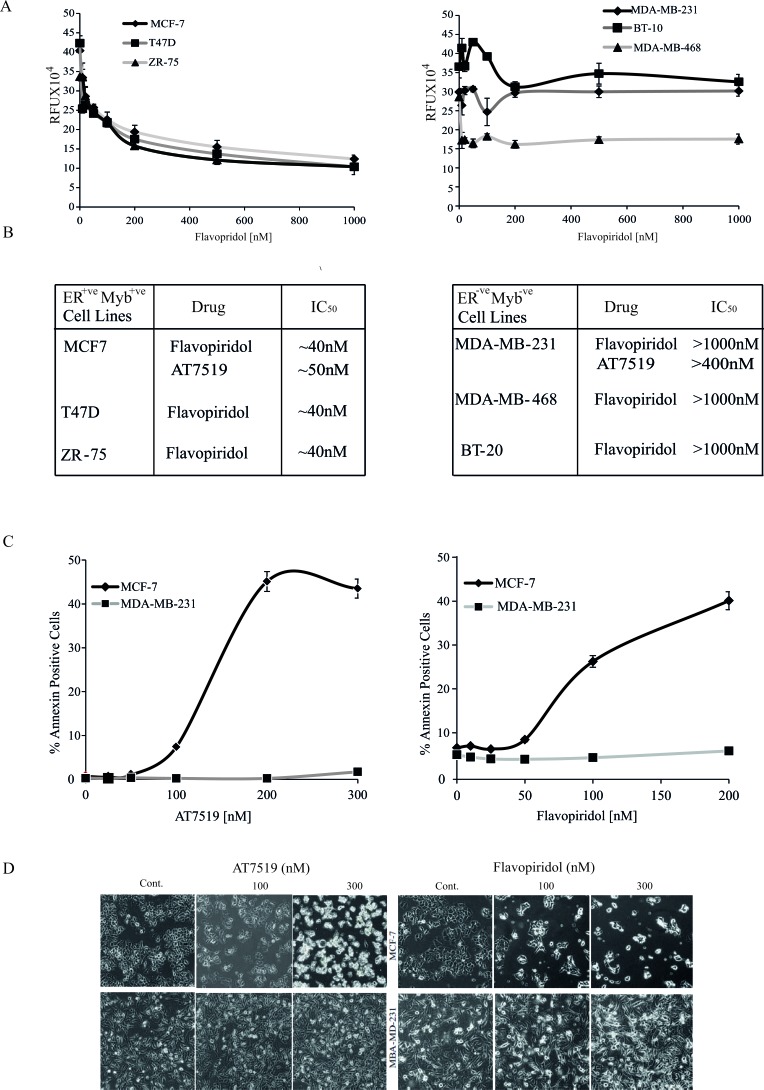
ER^+ve^ breast cancer cells are more sensitive to CDK9i than ER^−ve^ cells **A.** MCF-7, T47D, ZR-75 (ER^+ve^ MYB^+ve^; left panel) and MDA-MD-231, BT-10, MDA-MB-468 (ER^−ve^ MYB^−ve^; right panel) cell lines were incubated with Flavopiridol for 72h. Cell viability was estimated using a Resazurin fluorescence assay. Experiments were done twice in triplicate and the error bars indicating SD. **B.** Approximate IC50s were estimated from the data in **A.**. **C.** CDK9i induce apoptosis in ER^+ve^MYB^+ve^ cells. MCF-7 and MDB-MB-231 cells were incubated with either Flavopiridol or AT7519 as indicated for 48h. Apoptosis was estimated by AnnexinV staining followed by FACS analysis. **D.**. Phase contrast micrographs of cells from **C.** taken immediately before harvesting.

Since we previously showed that *MYB* expression is essential for proliferation and contributes to survival of ER^+ve^*MYB*^+ve^ breast cancer cells [4], we wanted to investigate the effect of CDK9*i* on the proliferation and viability of a panel of breast cancer cell lines. Therefore, MCF-7, T47D and ZR-75 cells were incubated with increasing doses of Flavopiridol for 72h. To examine potential correlation of any effects observed with *MYB* expression, we also used a panel of ER^−ve^*MYB*^−ve^ cell lines (MDA-MB-231, MDA-MB-468 and BT-10). It is clear that Flavopiridol has a dose-dependent effect on numbers of viable ER^+ve^*MYB*^+ve^ cells, with IC50 values of 40-50nM for each line (left panels of Figure 2A and 2B). In contrast, all 3 ER^−ve^*MYB*^−ve^ lines were quite resistant with IC50 values ≥ 1000nM. This selective sensitivity of ER^+ve^*MYB*^+ve^ cell lines to CDK9*i* was further confirmed using AT7519 (Figure 2B), for which the IC50 for MCF-7 was ∼50nM while for MDA-MB-231 it was > 400nM. In agreement with our observations, values of 40nM and 340 nM were reported by Squires et al. [19]for MCF-7 and MDA-MB-468, respectively.

We next wished to determine whether the effect of CDK9*i* on cell viability of ER^+ve^*MYB*^+ve^ breast cancer cells was associated with apoptosis and cell death. Therefore, MCF-7 or MDA-MB-231 cells were treated with either AT7519 or Flavopiridol for 48h. Cells were harvested and stained to estimate the proportions of total (AnnexinV^+ve^) and late apoptotic (AnnexinV and PI double-positive) cells (Figure 2C). In the presence of either drug, almost ten-fold more late apoptotic MCF-7 cells accumulated than was seen with MDA-MB-231. Microscopic examination of drug-treated MCF-7 and MDA-MB-231 cells at selected concentrations (Figure 2D) also clearly showed corresponding effects of these CDK9*i* on cell morphology and cell number. That these effects were largely due to CDK9 inhibition is reinforced by the observation that the MCF-7 cells were also much more sensitive to the highly selective CDK9 inhibitor BE-09-LN53 than were MDA-MB-431 cells (Supplementary Figure S3).

### Ectopic expression of *MYB* is sufficient to suppress CDK9 inhibitor-mediated induction of apoptosis

The selective effect of both drugs on ER^+ve^MYB^+ve^, compared to ER^−ve^MYB^−ve^ cells at relatively low concentrations implies they are acting on specific target genes or pathways in the former to induce apoptosis. Our published data and those in Figure 1 showed that transcription of *MYB* in ER^+ve^MYB^+ve^ breast cancer cells is regulated by the P-TEFb complex and suppressed by CDK9*i* [12]. This suggests that *MYB* inhibition is a potential mediator of the effects these drugs, particularly given that MYB directly regulates the anti-apoptotic gene *BCL2* [11].

**Figure 3:**
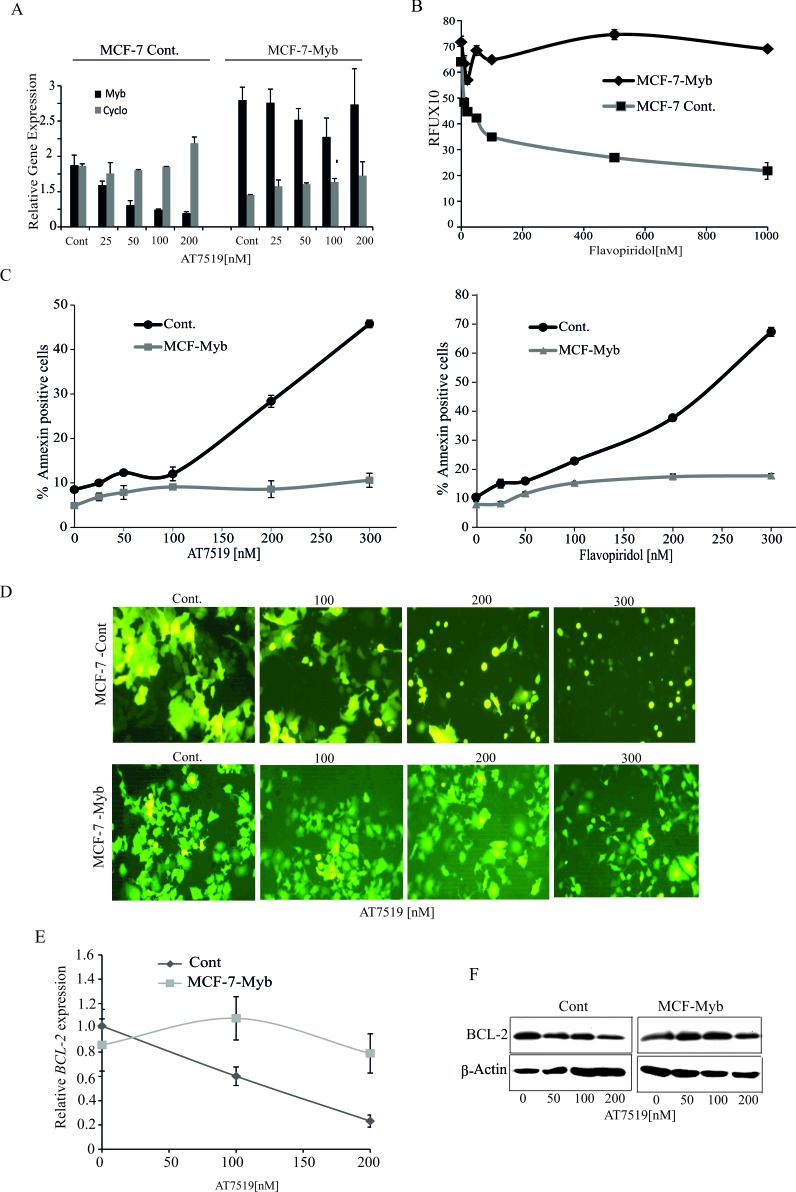
Ectopic expression of MYB is sufficient to overcome CDK9 inhibitor mediated apoptosis of ER^+ve^ breast cancer cells **A.** AT7519 treatment has no effect on MYB levels in ectopic MYB-expressing MCF-7 cells. MCF-7-Myb and vector control MCF-7-Cont cell lines were incubated with AT7519 at the indicated concentrations for 4h and harvested to measure the expression of MYB by qPCR, with *Cyclophilin A* (*PPIA*) as an internal control. **B.** Ectopically-expressed MYB promotes survival of MCF-7 cells in the presence of Flavopiridol. MCF-7-Cont and (MCF-7-Myb) cells were incubated with Flavopiridol for 72h before performing Resazurin viability assays. Experiments were carried out twice in triplicate **C.** Ectopic MYB expression promotes resistance to apoptosis induced by CDK9*i*. MCF-7-Myb and -Control cell lines were incubated with either AT7519 (left panel) or Flavopiridol (right panel) for 60h before harvesting for AnnexinV/PI staining and FACS analysis. **D.** Fluorescence micrographs of cells from **C.** taken prior to harvesting. (E and F) AT7519 downregulates *BCL-2* expression. MCF-7-Cont. and MCF-7-Myb cell lines were incubated for 4h or 16h with indicated dosages of AT7519 to determine the *BCL-2* gene **E.** or protein expression **F.**, respectively with β-actin gene and protein expression as internal and protein loading controls.

To address this, we used lentiviral vectors to establish a stable MCF-7 cell line expressing MYB (MCF-7-Myb), and control cell line with the corresponding empty pLV411vector (MCF-7-Cont). Both cell lines were treated with varying doses of AT7519 for 4h and then *MYB* levels were measured by q-PCR. Figure 3A shows that, as expected, *MYB* levels decreased in the control cells but were maintained in the MCF-7-Myb cells, because transcription from the lentiviral EF1A promoter is not primarily regulated by transcriptional elongation. Note also that the *MYB* level in the MCF-7-Myb line was less than twice the endogenous level in untreated control cells, i.e. *MYB* was not expressed at supra-physiological levels. Moreover, the AT7519 doses used did not adversely affect the cell's transcription machinery overall, since expression of *PPIA* (*Cyclophilin A*) was unperturbed. Similar results were obtained with Flavopiridol treatment (data not shown). Next, the two cell lines were treated with Flavopiridol for 72h and viable cells assessed by Resazaurin assay. As shown in Figure 3B, the control MCF-7 cells responded similarly to the parental cell line with a dose-dependent decrease in viable cells. However, a dramatic difference was observed with the MCF-7-Myb line, where Flavopiridol treatment remained ineffective up to 1000nM. Similarly, AnnexinV and PI staining showed that, as observed above, both drugs effectively induced apoptosis in control cells but not MCF-7-Myb cells (Figure 3C), which is also clear from the cell morphology where the normal flat epithelial shape of MCF-7-Cont cells changed to the shrunken, spherical appearance of apoptotic cells (Figure 3D, upper panels). Therefore, we conclude that maintenance of *MYB* expression can overcome the sensitivity of MCF-7 cells to CDK9*i*, which is in turn consistent with *MYB* down-regulation being a primary mediator of ER^+ve^MYB^+ve^ breast cancer cell killing by this class of drugs.

We previously showed that the anti-apoptotic protein *BCL2* is a direct MYB target gene in MCF-7 cells [11]. Thus it is possible that CDK9*i*-induced apoptosis may be mediated by the down-regulation of this gene. We therefore examined expression of *BCL2* in MCF-7-Cont cells and MCF-7-Myb cells following 4h treatment with AT7519. As shown in Figure 3E and 3F, drug treatment resulted in dose-dependent suppression of *BCL2* mRNA and protein in MCF-7-Cont cells. In contrast, no significant decrease was observed in the level of either protein or mRNA in MCF-7-Myb cells. These data strongly support the hypothesis that BCL2 down-regulation resulting from inhibition of *MYB* transcription contributes to the apoptotic effect of CDK9i on ER^+ve^MYB^+ve^ breast cancer cells.

### CDK9*i* induce G2/M cell cycle arrest and down-regulate *MYB* target genes *CCNB1* and *CCNE1*

**Figure 4:**
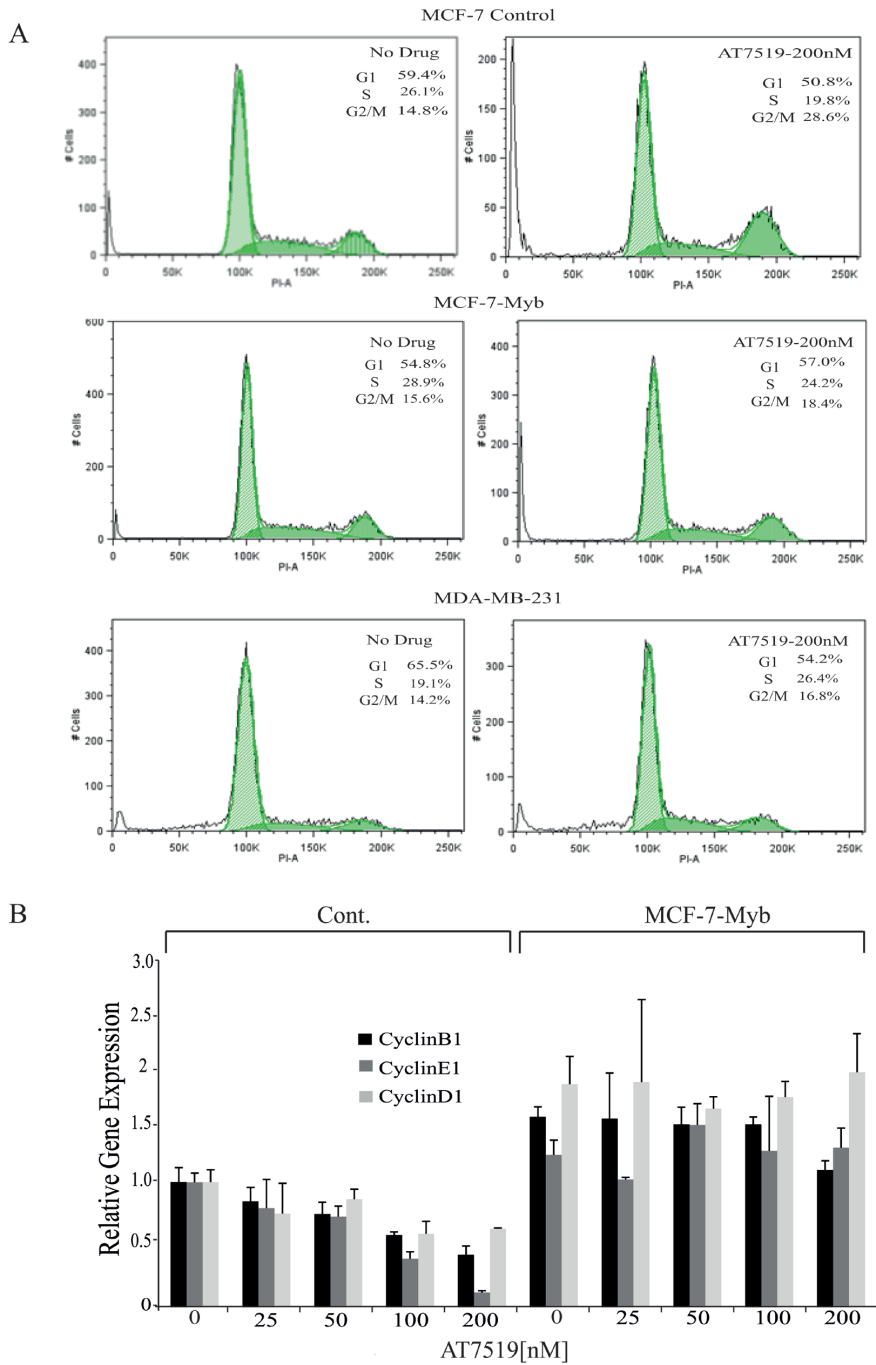
MYB target gene expression in and cell cycle analysis of MCF-7 cells with or without ectopic MYB expression **A.** Flow cytometric analysis of cell cycle progression by PI DNA staining in the indicated cell lines which were either untreated or treated with 200nM AT7519 for 48 hours. The percentage of cells in each cell cycle phase is also indicated in each panel. **B.** Expression of cyclin genes CCNB1, CCNE1 and CCND1 determined by qPCR in MCF-7-Cont and -Myb cells treated with the indicated concentrations of AT7519 for 48 hours. Expression of each gene is shown relative to the expression of it in untreated MCF-7-Cont. Experiments were performed in triplicate.

In addition to the apoptotic effects described above, it seemed possible that the decrease in viable cell numbers in ER^+ve^MYB^+ve^ breast cancer cells treated with CDK9*i* may also reflect direct inhibition of proliferation; this would be at least consistent with visual inspection (e.g. Figure 2D). We therefore analysed cell cycle progression in treated MCF-7-Cont, MCF-7-Myb and ER^−ve^MYB^−ve^ MDA-MB-231 cells with 200nM AT7519 for 48h (Figure 4A). As seen above, CDK9*i* treatment induced a significant amount of cell death, indicated here by increased sub-G1 DNA content, in control MCF-7 cells, but not in MCF-7-Myb or MDA-MB-231 cells. In addition, an approximately two-fold increase was also observed in the G2/M population in drug-treated MCF-7-Cont cells compared to untreated cells. At the same time, we observed a decreased number of cells in S phase. In contrast, little or no increase in G2/M cells was seen in either MCF-7-Myb or MDA-MB-231 cells.

These observations suggested *MYB* suppression by CDK9 inhibition was indeed affecting cell cycle progression both at the G1/S transition, as previously reported [22] and during the G2/M phases. MYB has been reported to directly activate the expression of *CCNE1* (in normal and transformed colonic epithelium [23] and *CCNB1* (in leukaemia and breast cancer cells; [24, 25]. We therefore examined expression of these genes and of *CCND1*, which is not a known MYB target, in MCF-7-Myb and control cell lines treated with AT7519. Expression of *CCNB1* and *CCNE1* was down-regulated with increasing doses of AT7519 in the control cell line but not in MCF-7-Myb cells, which is consistent with the regulation of these genes by MYB (Figure 4B). Surprisingly, the level of *CCND1* showed a similar pattern albeit to a lesser degree; it is conceivable that the increased level of *CCND1* in MCF-7-Myb cells reflects an indirect effect mediated by MYB's ability to enhance ER expression [26, 27].

### Role of *MCL1* in CDK9 inhibitor-induced killing of ER^+ve^ *MYB*^+ve^ breast cancer cells

**Figure 5:**
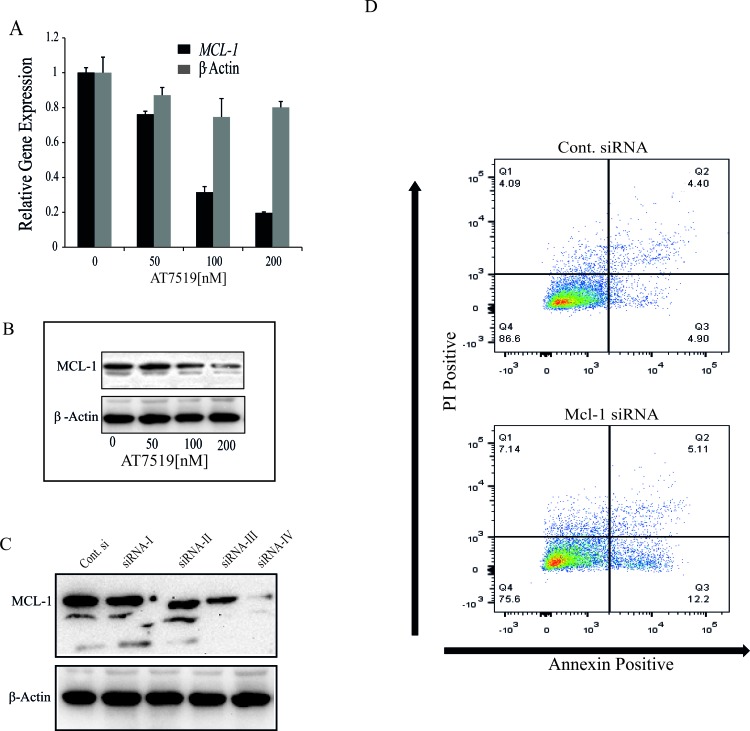
CDK9 inhibitor-mediated down-regulation of MCL-1 is not sufficient to induce apoptosis in ER^+ve^ breast cancer cells **A.** CDK9 inhibitor AT7519 downregulates the expression of MCL-1. MCF-7 cells were treated with AT7519 (0, 50, 100 and 200nM) for 4h and cells were harvested to estimate the gene expression by qRT-PCR. Expression of β-actin was used as an internal control and the relative expression of both genes were estimated using 18s-ribosomal gene expression as a loading control. **B.** MCF-7 cells were treated with AT7519, as indicated, for 16h and the expression of MCL-1 was determined by western blotting using β-acting expression as loading control. **C.** MCF-7 cells were transfected with four separate siRNA oligos (siRNA-I, II, III, IV) targeted to *MCL-1* and a control siRNA (Csi). Expression of *MCL-1* was estimated 72h after transfection by western blotting where β-actin was used as a loading control. **D.** siRNA-IV and Csi RNA were transfected with in MCF-7 cells and after 72h cells were harvested to analyse the induction of apoptosis by AnnexinV and PI staining followed by FACS analysis.

The data presented above all imply that *MYB* down-regulation, most likely acting through its target *BCL2*, is an essential contributor to apoptosis in MCF-7 cells by CDK9*i* such as Flavopiridol and AT7519. However, *MYB* suppression alone does not appear to be sufficient to induce a significant degree of apoptosis, as shown by our previous shRNA studies [4]. Given several studies showing an important role for *MCL1* in the resistance of breast cancer cells to apoptosis [13], and other reports that *MCL1* expression is suppressed at the level of transcriptional elongation by CDK9*i* in a range of cell types [28, 29], we decided to explore its role in the present system. We first confirmed that AT7519 induced rapid down-regulation of *MCL1* mRNA (Figure 5A and 5B), and then used siRNA to suppress its expression in MCF-7 cells (Figure 5C). However, even the most effective of the 4 siRNAs tested (siRNA-IV) induced only a small increase in the number of apoptotic (Annexin V-positive) cells (Figure 5D).

**Figure 6:**
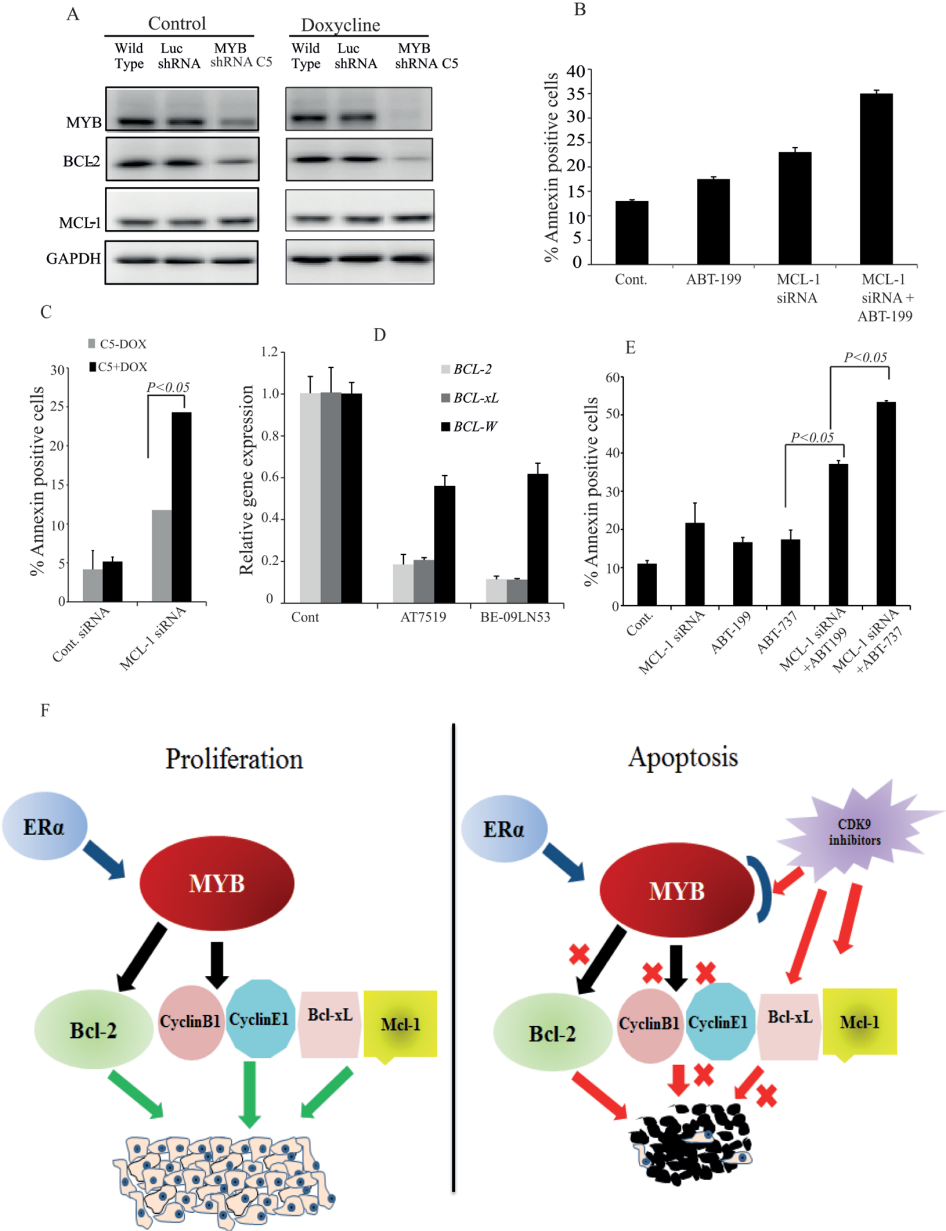
Maximal apoptosis induction in MCF-7 cells requires inhibition of the MYB - BCL-2 axis, MCL-1 and BCL-xL **A.** MYB down regulation affects BCL-2 expression but not that of MCL-1. The MYB shRNA cell line (MYB shRNA C5) and control shRNA cell line (Luc shRNA) along with wild-type MCF-7 cells were incubated without or with 5μg/ml Doxycycline for five days. Cells were harvested for western blot analysis of MYB, BCL-2 and MCL-1 using GAPDH as loading control. **B.** Both inhibition of BCL-2 activity and downregulation of *MCL-1* are required for apoptosis induction in MCF-7 cells. Cells were treated in triplicate with either 1μM ABT-199, transfected with *MCL-1* siRNA or both for 72h. Cells were harvested to estimate the amount of apoptotic cells by AnnexinV and PI staining followed by FACS analysis. **C.** The MYB shRNA C5 cell line was treated with or without 5μg/ml Doxycline for three days before cells were re-plated and transfected with *MCL-1* siRNA as described for **B.**. The mean (*n* = 3) percentage of AnnexinV positive cells determined by flow cytometry is plotted against each treatment group. **D.** Expression of *BCL-2, BCL-xL* and *BCL-W* measured by q-PCR following treatment with 300nM AT7519 or 200nM BE-09-LN53 for 4h. **E.** Simultaneous inhibition of BCL-2, BCL-xL and MCL-1 induced maximal apoptosis. MCF-7 cells were treated with either ABT-199 (2μM) or ABT-737 (2μM) alone or in combination with *MCL-1* siRNA transfection. Cells were harvested after 72h to determine the Annexin V positive cells. **F.** Model for the effects of CDK9i on proliferation and apoptosis of ER^+ve^MYB^+ve^ breast cancer cells. Survival and proliferation are driven by MYB targets BCL-2, CyclinB1, CyclinE1 and MYB-independent proteins BCL-xL and MCL-1. CDK9i induce apoptosis by inhibiting simultaneously the MYB-BCL-2 axis and MCL-1 and BCL-xL production.

We then considered that *MCL-1* might be required, even though not sufficient, to protect MCF-7 cells from apoptosis, since we found that CDK9 inhibitor treatment simultaneously inhibited expression of two major anti-apoptotic proteins, BCL-2 and MCL-1 (Figures 3 and 5). To explore this possibility, we treated MCF-7 cells with the specific BCL-2 inhibitor ABT-199 [30], MCL-1 siRNA-IV or both. Treatment with either ABT-199 or MCL-1 siRNA alone induced only a small increase in the number of apoptotic cells above control levels, while a substantially larger increase was observed when cells were treated with ABT-199 and MCL-1 siRNA-IV in combination (Figure 6B). These observations are somewhat in accord with those reported in [31], although we note that these authors used the ABT-737 compound which inhibits BCL-2, BCL-X_L_ and BCL-W (see also below). Nevertheless, the combined effect of BCL-2 and MCL-1 inhibition on the proportion of Annexin V-positive cells was consistently less than that seen with CDK9*i* (e.g. Figures 2C and 3C). We therefore wished to determine whether CDK9i had pro-apoptotic effects beyond those mediated by combined *MCL-1* and *MYB* downregulation.

To obtain further support for the proposition that inhibition of *MYB* expression enhanced apoptosis through downregulation of *BCL2*, we employed a stable cell line generated by transducing MCF-7 cells with a lentivirus carrying a doxycycline (Dox)-inducible *MYB* shRNA (C5) similar to that described previously [4], and a control cell line which carries shRNA targeting the firefly luciferase gene (Luc shRNA). As shown in Figure 6A, there was some leakiness in the *MYB* shRNA line, but nevertheless Dox induction reduced the level of MYB by more than 90% compared to parental or Luc shRNA MCF-7 cells. This was associated with a substantial reduction in the level of BCL-2. However, *MYB* down-regulation had no effect on the expression of MCL-1 (Figure 6A), consistent with the fact that *MCL-1* is not a known MYB target gene. We then asked whether the effect of *MYB* knockdown combined with *MCL-1* siRNA treatment result in a similar enhancement of apoptosis to that seen with combined *BCL-2* and *MCL-1* inhibition. Thus MCF-7 C5 cells were treated with or without Dox for 3 days, and then transfected with *MCL-1* siRNA. It is clear from Figure 6C that the combined effect on apoptosis of *MCL-1* and *MYB* knockdown was substantially greater than that of either alone, suggesting that simultaneous inhibition of both could mediate CDK9*i*-induced apoptosis.

However even this level of apoptosis was consistently less than that seen with CDK9*i* (e.g. Figures 2C and 3C), suggesting that CDK9 inhibition may directly affect other apoptotic regulators independently of *MYB*. To examine this possibility we measured the level of *BCL2L1* (*BCL-xL), BCL2L2 (BCL-W)* and *MCL1* mRNA following AT7519 or BE-09-LN53 treatment. Figure 6D shows that *BCL2L1* expression (in addition to that of *BCL2* and *MCL1*, as expected) was suppressed; however this was unlikely to be mediated by suppression of *MYB* because shRNA-mediated *MYB* knockdown did not affect *BCL2L1* expression (Supplementary Figure S4). Our observation is in agreement with reports of CDK9i suppressing *BCL2L1* expression in breast cancer cells (e.g. ref. [13]). Finally, we examined apoptosis induction by combined knockdown of *MCL1* and inhibition of both BCL-xL and BCL-2, using ABT-737 [32] (Figure 6E). ABT-737 alone, like ABT-199, increased apoptosis to ∼ 15% (5% above the untreated control), while as also seen in Figure 6B above, ABT-199 plus MCL-1siRNA increased this to ∼ 35%. However, *MCL-1* siRNA combined with ABT-737 resulted in a similar degree of apoptosis - about 55% - to that seen with the CDK9i. These data are consistent with the notion that apoptosis induction by CDK9i involves combined inhibition of MCL-1, BCL-2 and BCL-xL. Note that the overall effectiveness of CDK9i in killing ER^+ve^/MYB^+ve^ breast cancer cells is unlikely to be limited to 50% because (a) longer treatment increases the proportion of dead cells measured, and (b) the FACS-based assays used here are likely to underestimate the true proportion of dead cells (data not shown).

## DISCUSSION

Among the four major subtypes of breast cancer Luminal A, Luminal B (both ER^+ve^), HER2 positive, and triple negative/basal-like, the first two comprise more than 70% of breast cancer cases. ER^+ve^ breast cancers have the most favourable prognosis among the 4 groups and typically respond to endocrine therapies such as aromatase inhibitors, selective estrogen receptor modulators such as tamoxifen, and antagonists such as fulvestrant. However, resistance to endocrine therapy is common, with recent studies indicating almost 30% of patients receiving primary therapy either fail to respond or more commonly, develop secondary resistance [33]. Thus there is a pressing need for new therapies for this class of ER+ve breast cancers.

One approach is to target essential downstream effectors of estrogen/ER signalling in breast cancer. As detailed in the Introduction, *MYB* is one such effector [5, 7, 12, 34], and our previous data on the mechanism by which ER regulates *MYB* expression [12] have highlighted the possibility of targeting *MYB* with CDK9*i*. Here, we have explored this possibility and found that CDK9*i* down-regulate *MYB* expression and selectively induce apoptosis in ER^+ve^/MYB^+ve^ but not ER^−ve^/MYB^−ve^ breast cancer cells. In surviving cells, CDK9*is'* also induce a block at the G2/M phases of the cell cycle. Moreover, ectopic expression of MYB was sufficient to suppress CDK9 inhibitor-mediated apoptosis, strongly supporting the notion that *MYB* is a critical target of these inhibitors in ER^+ve^ breast cancer cells. We have also investigated the mechanisms by which CDK9*i* induce apoptosis and suppress cell cycle progression.

Our published work [12] showed that the CDK9 inhibitor DRB and Flavopiridol inhibit *MYB* expression by suppressing transcriptional elongation at the SL-dT pausing region in intron 1. In the present study we used additional CDK9i AT7519 [19] and BE-09-LN53 [21], and confirmed that these have similar effects on *MYB* transcription, and also selectively induce apoptosis in ER^+ve^/MYB^+ve^ breast cancer cells. This is significant because the latter compound shows a much better selectivity for CDK9 over other CDKs than do Flavopiridol and AT7519. For example, Flavopiridol inhibits CDK1, 2, 4, 5, 6, with IC50s of ∼40nM and CDK9 with IC50 ∼3nM, while BE-09-LN53 has IC50s for the former CDKs of >200nM and an IC50 of <0.4nM for CDK9 [21]. Thus the use of these compounds collectively indicates that effects on *MYB* transcription and cell killing are indeed due to targeting CDK9.

Nevertheless, the fact that CDK9 inhibition selectively induces apoptosis and G2/M cell cycle arrest in ER^+ve^/MYB^+ve^ breast cancer cells does not in itself show that *MYB* is a relevant target of these compounds; indeed expression of a substantial fraction of the genome is regulated by transcriptional pausing [35]. We have addressed this in part by showing that ectopic *MYB* expression can overcome the heightened sensitivity of in ER^+ve^/MYB^+ve^ breast cancer cells (compared to MYB^−ve^ cells) to CDK9*i*. It is noteworthy that the level of ectopic expression in our study was similar to that of the endogenous gene - the key difference is that expression of virally-encoded *MYB* is insensitive to CDK9 inhibition. Importantly we showed that CDK9i downregulate known MYB target genes with roles in suppression of apoptosis (*BCL2*) and cell cycle progression (*CCNB1* and *CCNE1*), and that this downregulation is also prevented by ectopic *MYB* expression. It remains to be explained, though, why we saw G2/M but not G1 arrest following CDK9 inhibitor treatment of MCF-7 cells, in view of the strong suppression of CCNE1 (encoding the G1/S cyclin E) and the fact that *MYB* knockdown by shRNA resulted in G1 arrest but not a discernible G2/M block (Drabsch 2007). It is possible that the G2/M effect reflects the combination of Cyclin B downregulation and the ability of AT7519 to inhibit CDK1 [20], which is also required for progression through mitosis. The reported IC50 for CDK1 is 210nM [20], so at least partial inhibition could have occured in the experiment shown in Figure 4.

It also became clear that the extensive induction of apoptosis by CDK9i we saw here was unlikely to be due to inhibition of *MYB* and *BCL2* expression alone. As we reported earlier [5] and confirmed here, we observed little or no apoptosis following shRNA-mediated *MYB* knockdown or treating cells with the BCL-2 inhibitor ABT-199. While it is well known (and reconfirmed here) that CDK9i downregulate *MCL-1* expression [36] [14], we did not observe significant cell killing by siRNA-mediated downregulation of *MCL-1* alone. However increased killing of MCF-7 cells was seen when *MCL-1* knockdown and BCL-2 inhibition (using ABT-199) were combined, implying that inhibition of both MCL-1 and BCL-2 is required to induce apoptosis in MCF-7 cells, and indeed a similar level of apoptosis was seen when both *MYB* and *MCL-1* were knocked down.

Nevertheless, the level of apoptosis seen with these combined treatments was consistently less than that seen with CDK9i, suggesting that CDK9 inhibition has additional pro-apoptotic effects. This notion is supported by our observation that CDK9 inhibition, but not *MYB* knockdown, reduces the level of *BCL2L1* expression, and that *MCL-1* knockdown combined with inhibition of both BCL2 and BCL2L1 by ABT-737 increased the level of apoptosis to a level similar to that obtained induced by CDK9i. In any case, our observations strongly support the proposition that apoptosis induction and killing of ER^+ve^MYB^+ve^ by CDK9i, as well as cell inhibition of cycle progression, is due to the combined direct inhibition of *MYB, MCL-1* and *BCL-xL* expression. These notions are illustrated in the model shown in Figure 6F and importantly provide an explanation for the relative insensitivity of ER^−ve^/MYB^−ve^ breast cancer cells to CDK9*i.*

Recently, Xiao et al [37] also reported that apoptosis of a series of breast cancer cell lines was enhanced by combined chemical inhibition of anti-apoptotic BCL-2 family members MCL-1, BCL-xL and BCL-2. Their results are broadly in agreement with ours as they used Navitoclax, a pharmacologically-optimised derivative of ABT-737 [38, 39], to inhibit BCL-2 and BCL-xL simultaneously and showed that this synergised with MCL-1 inhibition, although they did observe a somewhat greater loss of viability of MCF-7 cells following MCL-1 inhibition alone than we did here. Interestingly though, and in agreement with what would be predicted from our observations, they did not observe a significant synergistic effect of Flavopiridol and Navitoclax on these cells.

CDKs have received considerable attention as major targets for anti-cancer drugs, and more than 20 compounds that inhibit CDK9 and other CDKs have entered in different phases of clinical trials [40, 41]. The anti-proliferative and pro-apoptotic effects of these drugs on lymphomas and leukemias [40, 42, 43] have been pursued through preclinical development and clinical trial. However their effects on solid tumours, and particularly in breast cancers, have not been as extensively studied. One potential drawback with many of the current compounds is their activity against multiple CDKs - although this has also been proposed as a benefit - and consequently, the limited information regarding the precise mechanisms of their anti-tumour actions. Our present data together with our previously-published data [12], show the activity of CDK9*i* against ER^+ve^/MYB^+ve^ breast cancer cells and provide a mechanistic basis for this activity.

Our data also highlight the potential for development and use of these compounds, including highly selective CDK9*i*, in treating ER^+ve^ breast cancer through concurrent inhibition of *MYB, MCL-1* and other regulators of apoptosis. This will need to be pursued through preclinical models including cell line-based or patient-derived xenograft studies in mice. More broadly our data suggest that targeting transcription regulation of *MYB* with CDK9i might be a viable approach in other *MYB*-dependent cancer types, since *BCL-2* and/or other apoptotic regulators are also regulated by MYB in myeloid leukaemias, T-cell leukemias and colon cancers.

## MATERIALS AND METHODS

### Cell culture

MCF-7 cells were maintained in culture as a monolayer in DMEM supplemented with 10%FBS (Hy Clone^TM^,), 1mM sodium pyruvate, 0.1mM non-essential amino acids, 10μg/ml insulin, Penicillin-Streptomycin (Invitrogen). T47D and ZR-75 cells were maintained in RPMI containing 10%FBS, 10μg/ml insulin, Penicilin-Streptomycin. HEK293, MDA-MB-231, MDA-MB-468 and BT-10 cell lines were maintained in DMEM containing 10% FBS, and Penicilin-Streptomycin.

### Generation of MCF-7-MYB and *MYB* shRNA stable cell lines

Full length *MYB* cDNA was cloned in plv411vector is described in [5]. The *MYB* hairpins C5 (5′CTGATAATGCTATCAAGAACCACTGGA

TTCATGAGA TCCAGTGGTTCTTGATAGCATTATCAG) and the control hairpin shLUC (GTGCGTTGCTAGTA CCAACTTCAA GAGAGTTGGT ACTAGCAACG CAC) targeting Firefly Luciferase were cloned to the plv711 and Lentiviral particles were generated as described in Drabsch, et al. [4] Stable MCF-7 cell lines expressing MYB or harbouring *MYB* shRNA were selected on GFP fluorescence (MoFlo Astrios, Beckman Coulter). For shRNA mediated knockdown 2.5×10^6^ MCF-7C5 Myb or control MCF-7 Luc cells, were incubated 5 days without or with 5μg/ml Doxycyline. Subsequent expression of MYB proteins was analysed by western blotting.

### Cell viability assay

These assays were performed in 96-well plates with 1×10^3^ cells seeded one day before the drug treatment in a total volume of 0.2ml for 72h. Cells were harvested thereafter and assayed using Resazurin described [16] using FLUOstar Omega-BMG plate reader. All assays were performed in

### siRNA transfection

Four individual ON-Target plus siRNAs' targeted to human *MCL-1* were purchased from Dharmacon siRNA library. MCF-7 cells were seeded 1×10^5^ cells / well of a 12 well plate one day before transfection. Transfection experiment was carried out using Lipofectamin-2000 reagent following the manufacturers protocol (Invitrogen, USA). Cells were incubated for 72h and harvested for western bloting and apoptosis assay using Annexinn/PI staining.

### cDNA synthesis and q-PCR

Approximately 0.5×10^6^ cells were harvested 4h after drug treatment and RNA was isolated using an Invitorgen RNA isolation kit. First strand cDNA synthesis was carried out according to the manufacturers protocol (ABI High capacity cDNA synthesis kit, Thermofischer) from 2μg of RNA sample. To detect *MYB* intronic transcripts, the cDNA synthesis was carried out using a Superscript cDNA Synthesis Kit (Invitrogen) with Oligo (dT)_20_ primers following the temperature cycle 25°C for 10min (1X), 50°C for 75min (1X) and 85°C for 5min (1X). Primers used in q-PCR to detect gene expression, except *MCL*-1 (Forward 5′-AAGCCAATGGGCAGGTCT-3′, Reverse 5′-TGTCCAGTTTCCGAAGCAT-3′), β-actin exon (Forward 5′-AGAGCTACGAGCTGCCTGAC-5′and Reverse 5′-AGCACTGTGTTGGCGTACAG-5′), β-actin intron (Forward 5′-TTGCTTTTTCCCAGATGAGC-5′, Reverse 5′-GCTAAGTGTGCTGGGGTCTT-5′) were described earlier [4, 12]. Unless otherwise mentioned, all q-PCR experiments were performed in triplicate and the error bars indicating standard deviation (SD).

### Drug treatment

The CDK9 inhibitors Flavopiridol (Sigma, USA), AT7519 (Astex Pharma, UK) and BE-09-LN53 (Novartis, Inc. USA) were dissolved in 100% DMSO to make 1mM stocks. 0.25×10^6^ cells were seeded in each well of a 6-well plate one day before the drug treatment in a total volume of 2ml of culture medium. Cell were incubated at 37°C during the drug treatment and were harvested after 4h for RNA extraction or after 48h for AnnexinV/PI assay or otherwise mentioned.

### AnnexinV /PI staining assay

Cells were harvested, washed twice with PBS at 4°C and resuspended at 1×10^6^ cell/0.1ml in Annexin-binding buffer (10mM HEPES, 140mM NaCl, 25mM CaCl_2_, pH7.4). 2μl of Propidium iodide (PI, 0.1μg/ml stock made in annexin-binding buffer) and 4μl of the AnnexinV-Alexa 647 conjugate (BioLegend, USA) were added to the cell suspension and incubated for 15min at room temperature before they were analysed by FACS (LSR-II, BD Biosciences). If it is not mentioned, all apoptosis assays were performed in triplicate.

### Cell cycle analysis

Cells were harvested and esuspended in ice cold 1.0ml 70% Ethanol and incubated o/n at 4°C. Approximately 0.5×10^6^ cells were resuspended in 0.5ml of 1x PI staining buffer (1xPBS, 0.1% BSA, 0.1%RNaseA and 100μg/ml Propidium iodide) and incubated at room temperature for 30min before FACS analysis.

### Western blot

Approximately, 7.5×10^6^ cells were resuspended in a 0.2ml extraction buffer (50mM Tris.HCl, pH6.8, 2% SDS, 10mM sodium fluoride) and sonicated with 4 cycles on a Sonics Vibra Cell machine. Sonicated extracts were centrifuged at 20,000xg for 10min at 4°C and supernatants were collected for protein estimation using DC^TM^ Protein Assay Reagent (Bio Rad, USA). 50μg protein was loaded in each well of 10% SDS-PAGE gel and transferred to PVDF membrane (Thermofisher). Blots were incubated in primary antibody diluted in 1xTBS, 5.0% BSA (Sigma, USA), 0.1% Tween-20. Primary antibodies against MYB (Milipore), BCL-2 (Santa Cruz Biotech), MCL-1 (Cell Signaling Technology, USA) were used at 1:000 dilution and those against β-actin (Santa Cruz Biotech), RNA PolII (anti phospho Ser2 and Ser5, Cell Signaling Technology, USA) were used at 1:500. Blots were developed using chemiluminescent reagent (Thermo Scientific) and the signals were detected with a Versa Doc Molecular Imager (Bio Rad).

## SUPPLEMENTARY MATERIAL FIGURES


