# Supplementary material for: CDK9 inhibitors selectively target estrogen receptor-positive breast cancer cells through combined inhibition of MYB and MCL-1 expression
Source: Oncotarget. 2016 Jan 24;7(8):9069–83. doi: 10.18632/oncotarget.6997 (PMC4891027; doi:10.18632/oncotarget.6997)
Supplement: Supplementary file 1 [file oncotarget-07-9069-s001.pdf]

CDK9 inhibitors selectively target estrogen receptor-positive breast cancer cells through combined inhibition of *MYB* and *MCL-1* expression

Supplementary Material

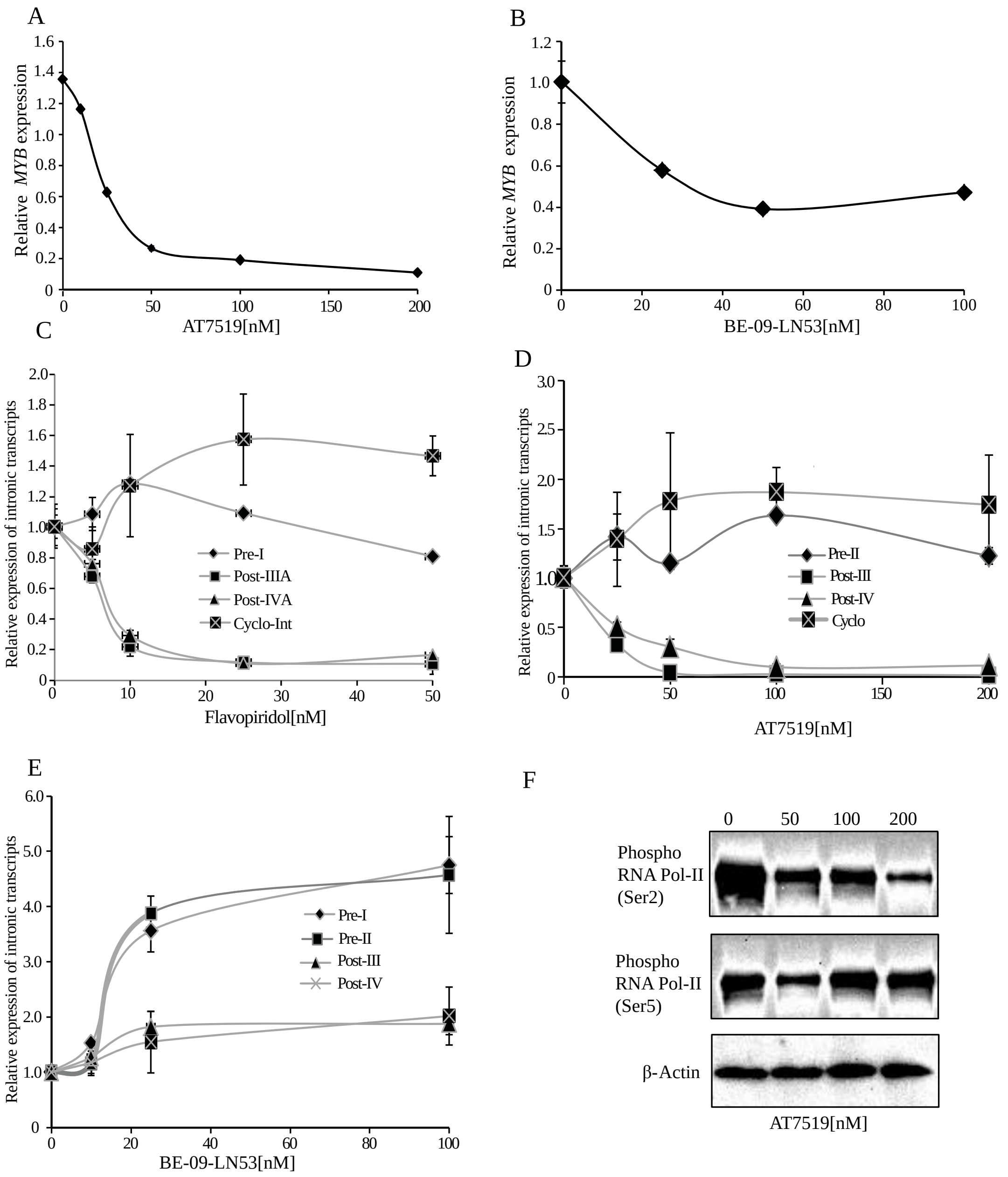

Supplementary Figure S1. CDK9 inhibitors downregulate *MYB* expression, impose transcriptional pausing, and inhibit RNA Pol II phosphorylation. MCF-7 cells were treated with CDK9 inhibitors AT7519 and BE-09-LN53 at different concentrations (0, 25, 50, 100 and 200µM) for 4h. Cells were harvested to check the expression of *MYB* by q-PCR

(A&B), and the short-lived intronic transcript **(C, D, and E)** by pre-post PCR as described in the Materials and Methods. **(F)** CDK9 inhibitors selectively downregulate the phosphorylation of ser-2 but not ser-5 residues of CTD of RNA pol II. MCF-7 cells were treated with increasing concentration of the CDK9i AT7519 for 24h. Pol II phosphorylation levels were determined by western blotting using anti-phospho (ser-2 or ser-5) RNA pol II-specific antibodies; β-actin was used as loading control.

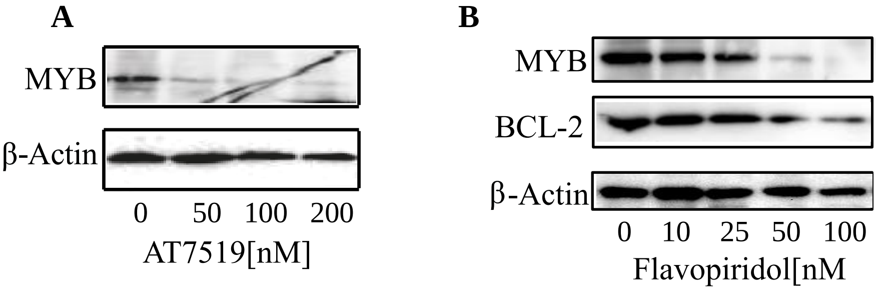

Supplementary Figure S2. CDK9*i* mediated transcription inhibition downregulates *MYB* and its target gene BCL-2 expression . MCF-7 cells were treated with different concentrations of CDK9 inhibitors AT7519 and Flavopiridol as indicated for 16h. Cells were harvested to check the expression of proteins by western blotting. **(A)** Approximately, 50μg of protein was loaded in each well and Western blotted with anti-MYB antibody. **(B)** Approximately, 100 µg of protein was loaded in each well and Western blotted with anti-MYB and anti-BCL-2 antibody. In both Western blots, β-Actin was used loading control.

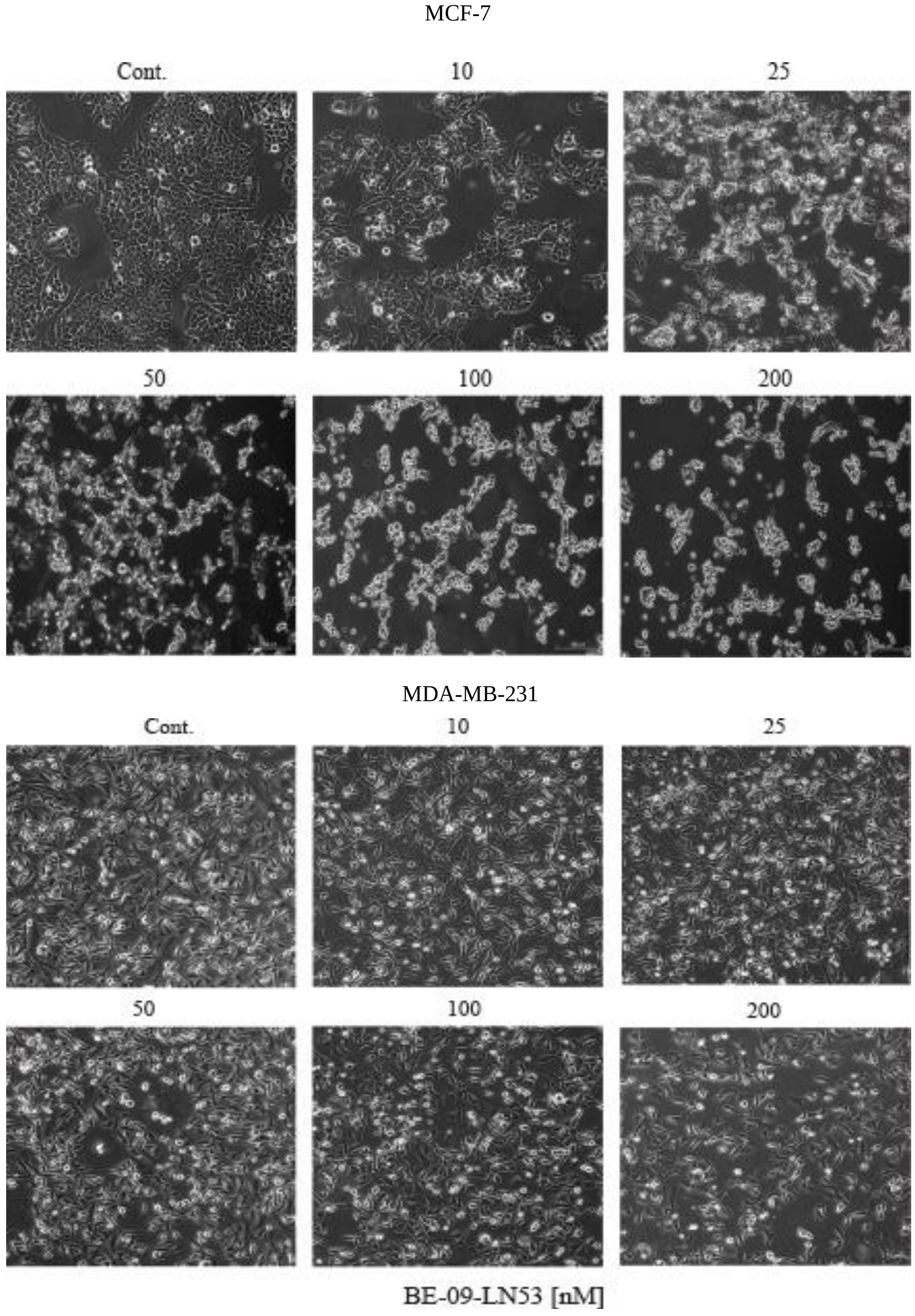

Supplementary Figure S3. Effect of the CDK9 inhibitor BE-09-LN53 on breast cancer cells. Phase contrast micrographs of MCF-7 (A) and MDA-MB-231(B) cells were treated with increasing concentrations of BE-09-LN53, as indicated at the top of each picture, for 48h.

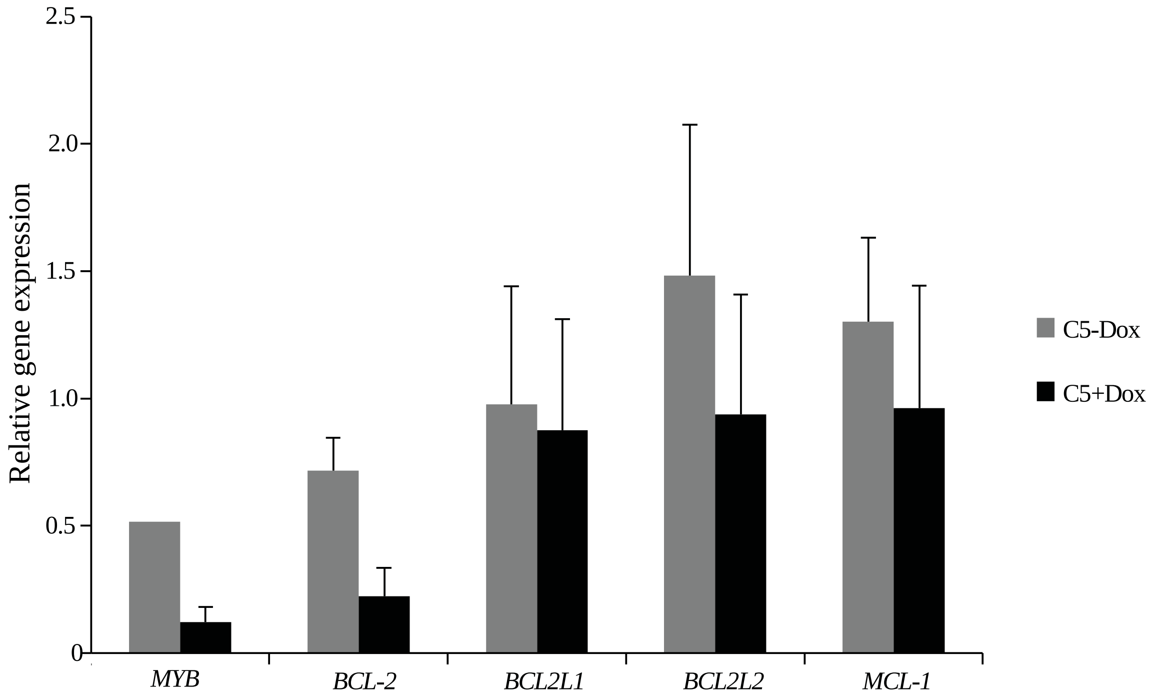

Supplementary Figure S4. Downregulation of *MYB* expression does not affect the expression of *BCL2L1* (*BCL-xL), BCL2L2 (BCL-W)* and *MCL1.* MCF-7 C5 cells were treated without or with 5µg/ml of doxycycline for five days. Cells were harvested to determine the expression of *MYB, BCL-2, BCL-xL, BCL-W* and *MCL-1* by qPCR. Gene expression was normalized using β-actin as an internal control.

A

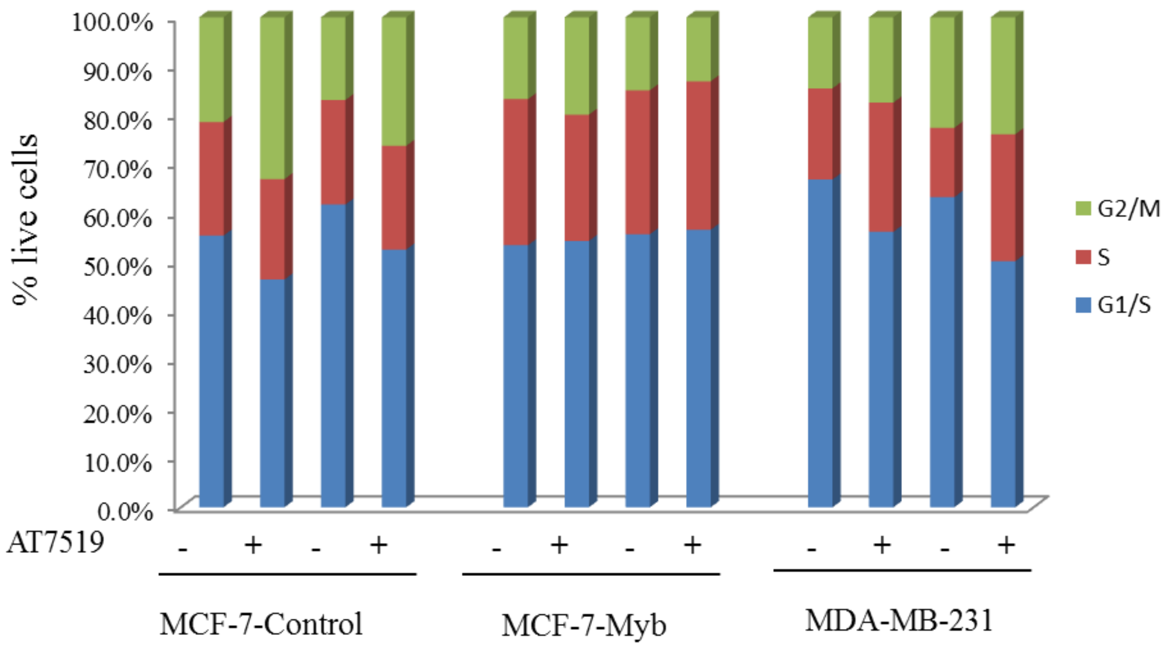
100.0%
90.0%
80.0%
70.0%
60.0%
50.0%
40.0%
30.0%
20.0%
10.0%
0.0%
% live cells
G2/M
S
G1/S
AT7519
- + - + - + - + - + - +
MCF-7-Control
MCF-7-Myb
MDA-MB-231

B

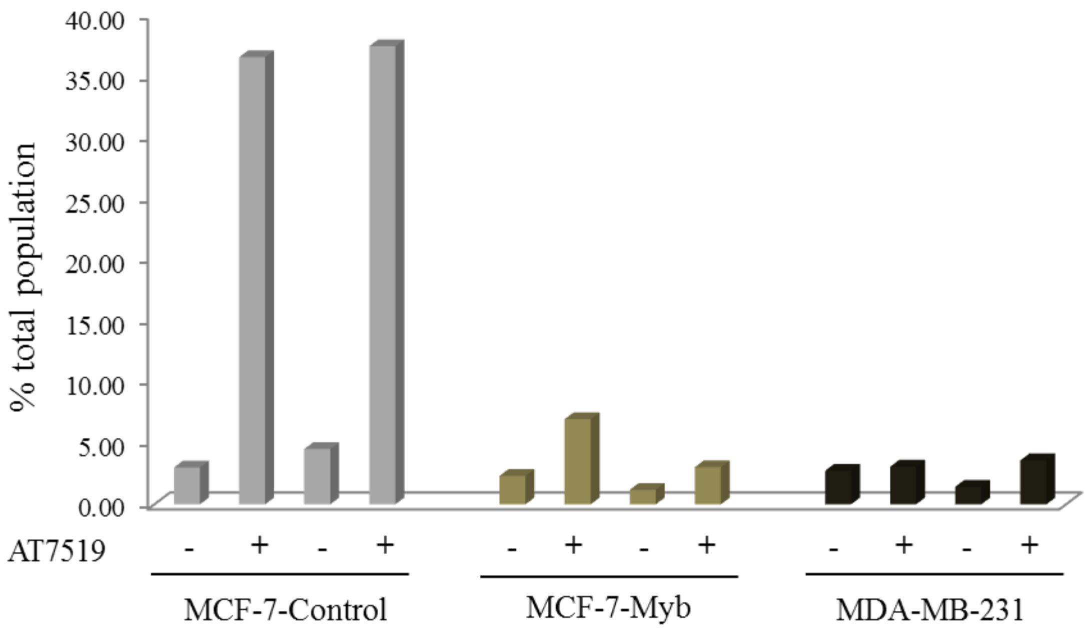
40.00
35.00
30.00
25.00
20.00
15.00
10.00
5.00
0.00
% total population
AT7519
- + - + - + - + - + - +
MCF-7-Control
MCF-7-Myb
MDA-MB-231

Supplementary Figure S5. CDK9*i* induced G2/M accumulation in MCF-7 but not in MDA-MB-231 and MCF-7-Myb cells. A.Two independent experiments were carried out with MCF-7, MCF-7-Myb and MDA-MB-231cells lines using 200nM of AT7519. Cells were harvested 48h to determine the effect of drugs on cell cycle progression. Amount of cells present at different stages of cell cycle was estimated as percentage of live cell population. **B.** Amount of cells present in Sub-2N population was estimated as percentage of total population.
